# Structural MRI-Based Predictions in Patients with Treatment-Refractory Depression (TRD)

**DOI:** 10.1371/journal.pone.0132958

**Published:** 2015-07-17

**Authors:** Blair A. Johnston, J. Douglas Steele, Serenella Tolomeo, David Christmas, Keith Matthews

**Affiliations:** 1 Division of Neuroscience, Medical Research Institute, Ninewells Hospital and Medical School, University of Dundee, Dundee, United Kingdom; 2 Advanced Interventions Service, Area 7, Level 6, South Block, Ninewells Hospital and Medical School, NHS Tayside, Dundee, United Kingdom; Leibniz Institute for Neurobiology, GERMANY

## Abstract

The application of machine learning techniques to psychiatric neuroimaging offers the possibility to identify robust, reliable and objective disease biomarkers both within and between contemporary syndromal diagnoses that could guide routine clinical practice. The use of quantitative methods to identify psychiatric biomarkers is consequently important, particularly with a view to making predictions relevant to *individual patients*, rather than at a group-level. Here, we describe predictions of treatment-refractory depression (TRD) diagnosis using structural T_1_-weighted brain scans obtained from twenty adult participants with TRD and 21 never depressed controls. We report 85% accuracy of individual subject diagnostic prediction. Using an automated feature selection method, the major brain regions supporting this significant classification were in the caudate, insula, habenula and periventricular grey matter. It was not, however, possible to predict the degree of ‘treatment resistance’ in individual patients, at least as quantified by the Massachusetts General Hospital (MGH-S) clinical staging method; but the insula was again identified as a region of interest. Structural brain imaging data alone can be used to predict diagnostic status, but not MGH-S staging, with a high degree of accuracy in patients with TRD.

## Introduction

Based on the 2010 Global Burden of Diseases Study, Whiteford, Degenhardt (1] estimated that mental and substance use disorders were the leading causes of years lived with disability worldwide. This is due to the chronic nature of psychiatric disorders and the typical onset of symptoms at a young age. Furthermore, depressive disorders were found to be the largest contributor to disability-adjusted life years, by some margin, within this grouping [1]. Major Depressive Disorder (MDD) is defined by persistent and disabling symptoms of low mood, anhedonia, hopelessness, guilt, low self-worth, poor concentration, lack of energy, suicidal thoughts and altered appetite and sleep [2, 3]. The diagnosis is elicited by clinical interview and mental state examination in the absence of robust established pathophysiological mechanisms or biomarkers. Remission rates with standard antidepressant drug treatments are only 30–40%, even when depression is accurately diagnosed and patient adherence is excellent [4–6]. The reliance on subjective symptoms in the absence of objective biomarkers, along with under-developed understanding of depressive endophenotypes may explain these treatment gaps.

It is, therefore, necessary to have a better understanding of the underlying mechanisms of depressive illness and to be able to identify objective biomarkers that can be used to make clinically-useful predictions for individual patients. Studies involving patients with so-called ‘treatment-resistant’ or, rather, treatment-refractory depression (TRD) are important because, by definition, these are the patients who do not respond well to available treatments and who experience chronic impairment, disability and social disadvantage. We have adopted the Massachusetts General Hospital—Staging (MGH-S) protocol [7] for quantifying treatment-resistance (similar to the Antidepressant Treatment History Form approach [8]). The crucial concept is of a ‘failed adequate trial’ of a treatment intervention; usually a medication. An adequate trial requires four criteria to be simultaneously satisfied: i) prescription of a given antidepressant medication for which there is evidence for efficacy with some patients with the diagnosis, ii) at a given minimum dose, iii) for a given minimum duration of time, iv) with reasonable certainty the patient took the medication as prescribed. If all four criteria are not met then the treatment does not constitute an ‘adequate’ trial. If all criteria are satisfied and the patient does not respond to treatment, then this is conceptualised as a ‘failed adequate trial’. Essentially, the total number of failed adequate trials (with some weightings to the scoring) constitutes a numerical measure of treatment resistance for an individual patient.

Whilst patients who are highly treatment refractory tend to experience chronic and enduring illness (evidenced by e.g. high Montgomery-Åsberg Depression Rating Scale (MADRS) or Beck Depression Inventory-II (BDI) scores) and functional impairment, they are not necessarily “in episode” continuously. For example, an individual patient can have shown minimal or no response to several ‘adequate’ medication trials and psychological therapy before finally responding to a course of electroconvulsive therapy. Such a patient would still be considered to fall within the category of treatment refractory despite the improved current health status. Treatment-resistance is, therefore, regarded as an enduring propensity to be resistant to antidepressant treatment and to be at increased risk of further episodes on the basis of previous clinical features. This can be considered as analogous to, for example, epilepsy, where a patient does not cease to have a propensity to epileptic seizures despite no recent seizure activity, particularly where seizure control has been difficult to achieve. This conceptualisation has the advantage of avoiding a conflation of concepts of current illness severity (e.g. MADRS) and treatment-resistance (e.g. MGH-S).

Ultimately, improved understanding of the underlying mechanisms contributing to TRD could facilitate more effective targeting of drug discovery research, substantially impacting on chronic disability worldwide. However, there are few studies of this important clinical population [6]. In the imaging studies that have focused on TRD, reduced hippocampal volume has been consistently implicated. Studies by Shah and colleagues reported reduced grey matter density in the left temporal cortex, including the hippocampus [9, 10], in TRD patients compared both with controls and recovered MDD patients. A more recent volumetric study reported that both TRD and treatment-resistant schizophrenia patients showed reduced hippocampal volumes compared with controls [11]. The volume of the entorhinal cortex, a structure with strong connections with the hippocampus, has also been reported to be significantly reduced in female patients with TRD compared with controls, but not in the corresponding male comparison [12].

Whilst there have been only a few studies specifically investigating patients with TRD, there are many studies which have reported *group-level* differences in brain structure between patients with MDD (non-TRD) and healthy controls; a majority of which have reported regional reductions in grey matter volume compared with healthy controls [10, 13–15]. MDD patients, compared with healthy controls, have been reported to show decreased grey matter in one or more meta-analysis in: the bilateral rostral anterior cingulate cortex; putamen; caudate; insula; globus pallidus; thalamus; hippocampus; and diverse areas within the frontal lobes [14, 16, 17]. Kempton *et al*. [17] also reported that MDD subjects had larger lateral ventricular and CSF volumes compared with controls. The amygdala and thalamus were reported to show no differences between groups by Kooschijn *et al*. [14], but the latter was found to be decreased in MDD patients in the more recent meta-analysis by Kempton *et al*. [17].

Regions identified using voxel-based morphometry (VBM) provide information about group-level differences. By contrast, techniques based on machine learning, such as support vector machines (SVM), which include additional techniques such as feature selection, can determine which brain regions consistently differ between groups in order to produce an accurate individual subject classifier. Although no reported study has used machine learning-based techniques to classify individual subjects with TRD, a number of studies have used these techniques to classify MDD patients and healthy controls ranging between 68–90% accuracy [18–20]. The highest accuracy reported was obtained by Mwangi and colleagues [20], who successfully classified structural MR images between MDD patients and healthy controls using images obtained over two scanning centres. In that study, grey matter reductions were identified in MDD participants compared to controls in the dorsolateral prefrontal cortex, medial frontal cortex, orbitofrontal cortex, temporal lobe, insula, cerebellum and posterior lobe. Identification of a pattern of brain abnormalities that is able to accurately predict TRD vs. healthy controls in individual subjects using machine learning-based techniques could increase the understanding of and elucidate the mechanisms of TRD. Similarly, identification of brain regions that support prediction and correlate with, for example, the MGH-S staging method, could be used to define novel biomarkers relevant to treatment non-response.

Another aim of the study was to investigate whether it was possible to make accurate *individual* predictions of symptom severity scores derived from the self-rated BDI and/or the clinician-rated MADRS and 17-item Hamilton Depression Rating Scale (HDRS_17_) using the same TRD participant structural images. Mwangi and colleagues previously used structural MR images to predict MDD illness severity [21]. In that study, they found that it was possible to predict BDI scores from whole-brain structural MRI scans [21].

Therefore, the primary aims of the present study were to:

1. classify accurately TRD participants and never-depressed controls on the basis of structural MRI scan data alone,
2. investigate whether the level of treatment resistance derived from the Massachusetts General Hospital (MGH-S) staging method [7] could be accurately predicted in the patient group,
3. investigate whether symptom severity scores derived from the self-rated BDI and/or the clinician-rated HDRS_17_ and MADRS could be accurately predicted in the patient group.

## Materials and Methods

Structural T_1_ weighted scans and contemporaneous clinical ratings were obtained at the Clinical Research Centre, Ninewells Hospital and Medical School in Dundee, UK. The study was approved by the East of Scotland Research Ethics Service. Written confirmation of informed consent was obtained from all volunteers.

### Participants

Twenty adults with a lifetime history of TRD, but who did not necessarily meet criteria for diagnosis of depressive episode *at time of scanning* were recruited from the Advanced Interventions Service in Dundee. All TRD participants had experienced lifetime and/or current chronic episodes of depression (>24m). Diagnoses were made by experienced clinicians according to DSM-IV criteria using the MINI PLUS (version 5.0) interview schedule [22]. The level of treatment non-response was based on detailed case note review and was scored using the MGH-S [7]; a clinical staging method for evaluating ‘treatment resistance’ that assigns points for ‘adequate’ trials (i.e. exceeding a minimum dose and duration of a given medication) of antidepressant medication. Scoring incorporates optimisation of antidepressant dose, antidepressant combinations and treatment augmentation strategies.

Study exclusion criteria included potentially confounding clinical features including: other primary psychiatric disorder (e.g. bipolar disorder, personality disorder or psychotic illness); history of current or previous substance misuse or significant head injury. Eighteen TRD participants were being treated with one or more anti-depressant medications at time of scanning (venlafaxine (6), sertraline (3), trazodone (3), citalopram (2), fluoxetine (2), isocarboxazid (2), mirtazapine (2), L-tryptophan (1), phenelzine (1), tranylcypromine (1)). In addition, 7 TRD participants were being treated with anti-psychotic medications (quetiapine (6) and chlorpromazine (1)). Three TRD participants were being treated with lithium augmentation.

Twenty-one healthy, never-depressed controls were recruited (mostly from partners, relatives and friends of TRD participants) and underwent clinical screening using the same semi-structured interview schedule and questionnaires. None of the controls had a history of current, or previous, psychiatric or neurological disorder, and all control participants were medication-free.

All participants had a predicted premorbid Full Scale Intelligence Quotient above 106 (one control was not assessed for IQ) as estimated by the National Adult Reading Test [23]. Handedness was assessed using the Edinburgh Handedness Inventory [24]. All subjects were right-handed with the exception of two left-handed participants in the control group and one and three ambidextrous participants in the control and patient groups respectively. Handedness was unknown in one participant from each group.

### Image Acquisition

Structural whole-brain images were acquired using a 3T Siemens Magnetom TrioTim syngo scanner using a T1-weighted MP-RAGE (magnetisation-prepared rapid acquisition gradient echo) sequence with the following parameters: TR = 1900 ms, TE = 2.64 ms, flip angle = 9°, FOV = 200 mm, matrix = 256 x 256, 176 slices, voxel size 0.8x0.8x1 mm, slice thickness 1 mm.

### Image Pre-processing

All scans were visually inspected for gross artefacts and particular care was taken to identify motion artefacts which appeared as blurring or ‘ghosting’ [25]. All scans were free from such artefacts and were included in the analysis.

Pre-processing was performed using SPM8 [26]. The procedure involved segmentation of T_1_ weighted images into separate grey matter, white matter and CSF compartment images and normalisation of the grey matter segmented images towards to the default SPM8 anatomical template. The resultant images were smoothed with an 8 mm full-width at half-maximum (FWHM) Gaussian kernel.

### Individual Subject Classification

Individual subject classification was implemented in Matlab (The Mathworks Inc.) using a SVM toolbox [27] with a Gaussian kernel [28] and custom Matlab scripts.

SVM analysis consisted of two stages: training the classifier, then testing the accuracy using data not used for training. Within-study replication (‘leave-one-out’ cross-validation, LOOCV) was used for training, meaning N-1 subjects are used as training data and the left out subject is predicted using the trained classifier, with **all subjects** used as testing data at some stage [29]. The SVM parameters were selected on the basis of training stage accuracy to avoid mixing prediction data with testing data which would result in inflated accuracy estimates.

Feature selection was used to identify brain regions supporting predictive *classification*. The feature selection method chosen during the SVM classification was a standard t-test, as implemented in the SPM toolbox. A t-test between the patient and control groups *within each training set* was performed during LOOCV (i.e. the subject being classified was not included in the t-test) with significance defined as p<0.05 at a whole brain, family-wise error corrected level [30]. The z-scores at each of the significant voxels were then ranked during LOOCV and the threshold, whereby voxels with z-scores above this threshold would be included in the classification, was optimised at the same stage as the SVM parameter selection. As the features selected differed slightly for each iteration of LOOCV, we report the sum of each feature selection map from each iteration, indicating which brain regions are most consistently selected for prediction of the ‘left out’ test dataset.

### Group-level Differences

In order to compare the regions identified during the classification with a more conventional group-level analyses, we performed a standard t-test on *all subjects*, as implemented in the SPM toolbox. This comparison demonstrates that the feature selection method, which involves selecting a threshold during cross-validation, corresponds to a more stringent threshold than using a conventional t-test, but otherwise the results are very similar. For the conventional *group*-level VBM analysis, the null hypothesis of no difference in brain structure between patients and controls was tested using an unpaired t-test as implemented in SPM8. Significance was defined as p<0.05 at a whole brain, family-wise error corrected level, with the simultaneous requirements for voxel threshold and minimum cluster extent identified using a popular Monte-Carlo method [30].

### Individual Subject MGH-S and Severity Score Predictions

Individual subject predictions of MGH-S and severity scores was implemented in Matlab (The Mathworks Inc.) using the Relevance Vector Regression (RVR) function within the PRoNTo toolbox [31, 32] and custom Matlab scripts. Similar to the SVM analysis, within-study replication (LOOCV) was used for training [29].

The RVR parameters were selected on the basis of a combination of three standard statistical variables: the root-mean square error (RMSE), the mean absolute error (MAE) and Pearson’s correlation coefficient (R) as calculated using the gfit2 toolbox [33]. The Shapiro-Wilk test was used to test whether each continuous variable was normally-distributed prior to using RVR (a requirement when using the Pearson correlation coefficient). The MGH-S and symptom severity scores all met this requirement.

The RVR predictions were performed on scans from TRD participants only, both with and without feature selection. The feature selection methods investigated and the corresponding results are reported in the S1 File.

### Group-level Correlations

Similarly, the *group*-level regressions were performed as implemented in SPM8 using TRD participant data and both the MGH-S and symptom severity scores (according to the HDRS_17_, MADRS and BDI-II, reported in the S1 File). Significance was defined as p<0.05 at a whole brain, family-wise error corrected level, with the simultaneous requirements for voxel threshold and minimum cluster extent identified using a popular Monte-Carlo method [30].

## Results

### Participant Characteristics

Age and estimated pre-morbid IQ (t-test, p>0.1, excluding the one control subject who failed to complete the testing) and gender distribution (as assessed by a chi-square calculation) did not differ significantly between groups. TRD participants mean ± SD age was 51.8 ± 11.2 years and mean ± SD estimated IQ was 122.8 ± 4.7. The control group mean ± SD age was 46.1 ± 14.0 years and the mean ± SD estimated IQ was 122.8 ± 5.8.

The average MGH-S score of the TRD participants was 13.3, which is consistent with a previous report of patients attending the Advanced Interventions Service (15.5) and should be noted as significantly higher than patients with depression treated in UK secondary care (5.3); and primary care (0.5) [34]. Indeed, this compares to the average MGH-S score for US patients enrolled in a speciality clinic, who had mean ± SD MGH-S scores of 1.6 ± 1.2 [35]. The average HDRS_17_, MADRS and BDI illness severity rating scores in the TRD group were 16.1, 22.5 and 32.2 respectively, reflecting group-level depression severity at time of scanning to be in the mild-moderate range. These results are summarised in Table 1.

**Table 1:** Clinical descriptors for the TRD and healthy control groups in the structural MRI analysis. Variables are shown as mean (standard deviation).

	MDD	Controls	
Age	51.80 (11.23)	46.14 (13.97)	n.s.
IQ	122.75 (4.71)	116.95 (27.38)	n.s.
Female/Total*	15/20	15/21	n.s.
HDRS_17_	16.10 (5.58)	0.48 (0.93)	<0.001
MADRS	22.50 (7.97)	0.48 (1.03)	<0.001
BDI	32.20 (11.38)	0.43 (0.87)	<0.001
MGH-S	13.25 (10.49)	N/A	N/A

*chi-square test with other tests being t-tests.

Two TRD participants were in remission at the time of scanning (as assessed through the MINI PLUS interview schedule and clinical ratings). Using a previously described scheme for categorisation of severity [36], HDRS_17_ scores were categorised into approximate levels of severity. Using this approach, one participant was classed in the “very severe” range, three in the “severe” range, ten in the “moderate” range, five in the “mild” range and one in remission at the time of scanning. Using alternate severity ranges as defined by Zimmerman, Martinez [37], two participants were classed in the “severe depression” range, seven in the “moderate” range, ten in the “mild” range and one in remission at the time of scanning. Using either categorisation, TRD participants had a wide range of symptom severity scores at the time of scanning.

### Individual Subject Classification

A Gaussian SVM was used and feature selection was implemented using t-tests with a variable threshold that was optimised during cross-validation. The analysis using grey matter images resulted in an individual subject predictive accuracy of 85% (sensitivity 0.85, specificity 0.86, χ^2^ = 17.7, p <0.0001).

**Fig 1:**
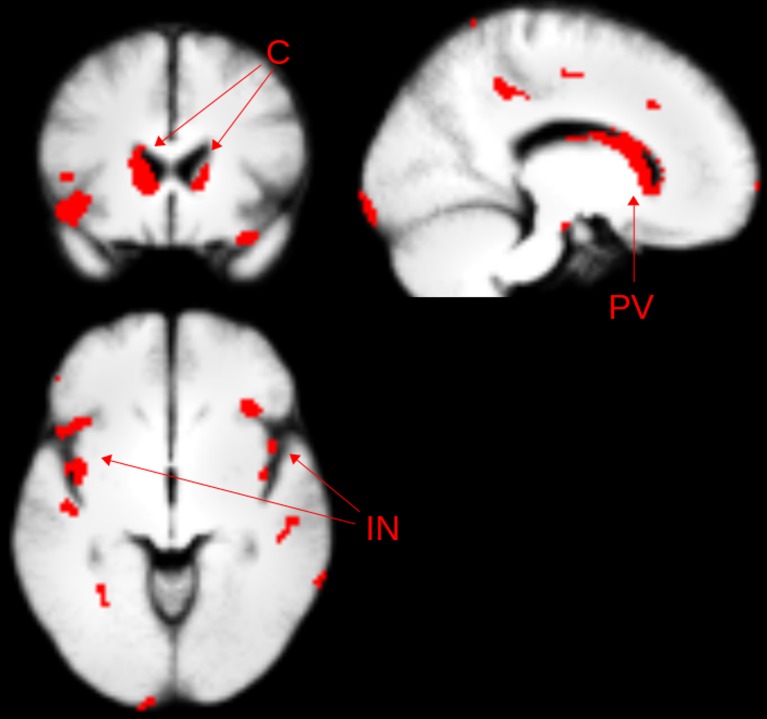
Feature selection (Gaussian SVM) identified brain regions in *grey* matter. PV—periventricular grey matter; C—caudate; IN—insula.

Grey matter reductions in the caudate, insula, and periventricular grey matter supported individual prediction at an accuracy of 85%. Regions used in the classification are shown in Fig 1 and Table A in S1 File.

### Group-level Differences

**Fig 2:**
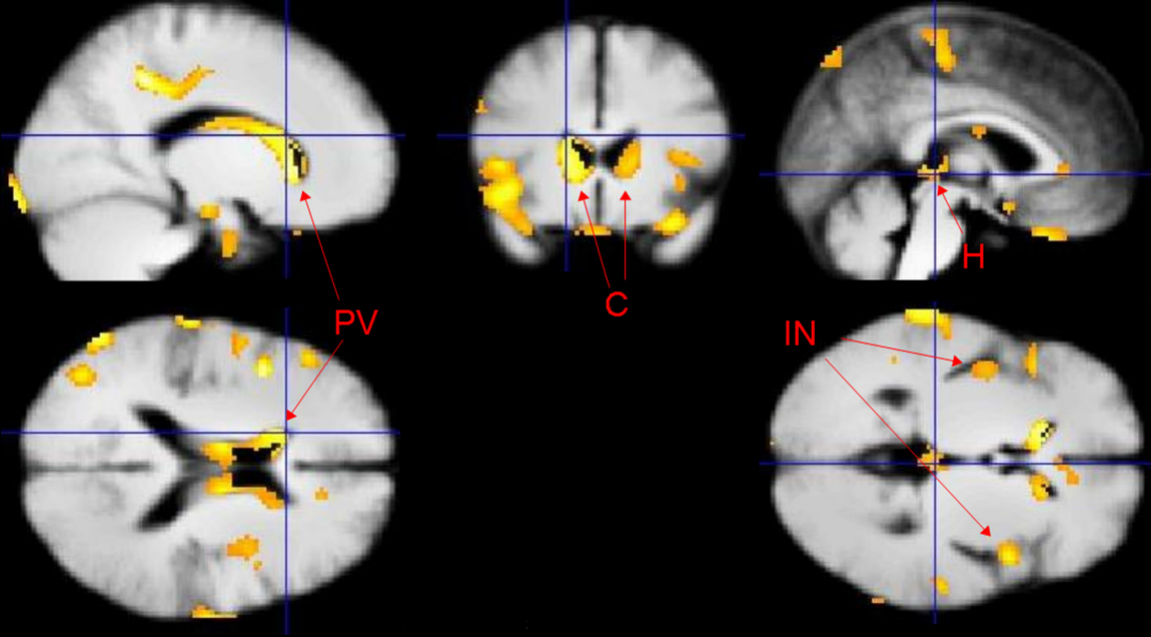
*Group*-level *grey* matter *reductions* in patients with TRD compared with healthy matched controls. PV- periventricular grey matter, C—caudate reductions, H—habenula and IN—insula.

The brain regions identified using feature selection overlapped to a great extent with the results of the VBM group-level analysis (p<0.05, whole brain level significance) as t-tests were used in both cases (although the t-tests used during feature selection *did not include the subject being classified* and not all significant voxels from the t-tests were used in the prediction as it was thresholded). In the VBM analysis, mostly grey matter *reductions* were identified in the TRD participants, but a small number of increases were also found in the posterior cerebellum and middle frontal gyrus. As shown in Fig 2 and Table B in S1 File, the largest grey matter *reductions* were found in the caudate, insula, and periventricular grey matter. In addition, TRD patients also had significantly reduced grey matter volumes in a region identified by Lawson, Drevets [38] as corresponding to the habenula. There were no significant group differences detected in the hippocampi.

**Fig 3:**
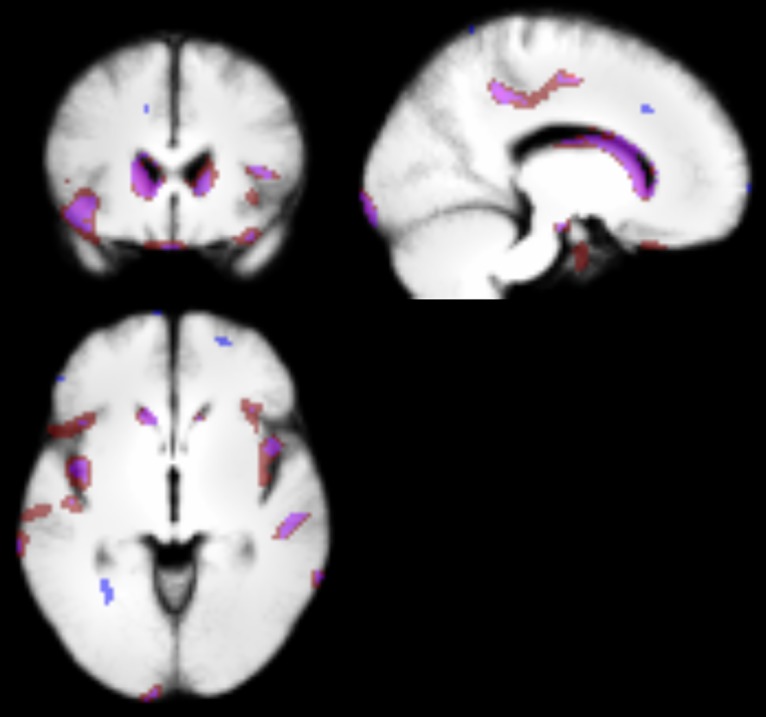
Overlapping grey matter regions between features selected during classification (purple/blue) and regions selected in the VBM analysis (red/purple).

Whilst the majority of the regions identified in the classification overlapped with the VBM results, a few voxels did not overlap either due to the thresholding of the t-tests during feature selection or differences in the t-test due to the left out subject during cross-validation. The overlap between the VBM results and the results from the feature selection is shown in Fig 3.

### Individual Subject MGH-S and Severity Score Predictions

A linear kernel RVR was used to predict symptom severity scores (HDRS_17_, MADRS and BDI) using the whole brain (no feature selection) MRI images from the TRD participants only. There was a significant correlation between the true and predicted HDRS_17_ scores (RMSE = 4.6963, MAE = 3.6212, R = 0.50712, p = 0.02). A significant correlation was also identified between the true and predicted MADRS scores (RMSE = 6.8328, MAE = 5.441, R = 0.4822, p = 0.03). The best-fit line between true and predicted scores for both the HDRS_17_ and MADRS predictions is shown in S1 Fig. and the highly distributed brain regions that provided the highest weightings are shown in S2 Fig. The most common regions to be used in both predictions were those along the cingulate gyri. However, the BDI scores could not be predicted in individual TRD participants using this approach. The results when feature selection was investigated is included in the S1 File.

Using the same method that was able to predict various symptom severity scores, it was not possible to predict MGH-S scores in individual subjects, irrespective of whether feature selection was used.

### Group-level Correlations

Grey matter volume did not correlate strongly with previous medication exposure (as assessed through the MGH-S scores). Indeed, there were no positive correlations with MGH-S scores, but the insula and various small cortical regions showed a negative correlation (as shown in S8 and S9 Figs and Table C in S1 File). Hence, TRD participants with greater ratings of refractoriness to treatment showed reduced grey matter volume in these regions.

The insula was previously identified in the VBM analysis as one of the regions which was significantly reduced in volume in TRD participants compared with healthy controls. However, as MGH-S scores did not correlate with any of the symptom severity scores (HDRS_17_, MADRS or BDI), it is reasonable that we did not find any overlap between brain regions that correlated with symptom severity (reported in the S1 File, S4–S7 Figs and Tables D-F in S1 File). Furthermore, adding MGH-S as a covariate in the HDRS_17_ regression (and vice-versa) made no substantial alterations to the results.

## Discussion

The high classification accuracy (85%) achieved in this study demonstrates that there are consistent patterns of structural brain abnormalities in patients with histories of TRD, including those with mild symptom burden, or in full clinical remission, at time of scanning when compared with healthy controls. These patterns were identified using feature selection and key regions included the caudate, the insula and periventricular grey matter—each of which have previously been reported to be reduced in previous group-level meta-analyses focusing on MDD [14–17]. The insula appears to represent an important brain region in TRD as it was found to correlate with MGH-S scores and was identified in the TRD vs. healthy control prediction. The insula has previously been implicated in the pathophysiology of depression as it has been associated with, among other things, emotional processing [39, 40] and human awareness and subjective experience [41]. As with our study, the volume of the insula was reduced in several other studies of patients with depression [20, 40, 42, 43]. Furthermore, these insula changes may be associated with clinical course and prognosis as Soriano-Mas and colleagues reported that insula volume was correlated with the number of relapses during a follow-up period and, along with the hippocampus and lateral parietal cortex, reduced volume of the left insula predicted a slower recovery [43]. The insula has also been reported to have decreased connectivity in TRD compared with treatment-sensitive depression [44].

The failure to accurately predict MGH-S scores in individual subjects may be due to the difficulties and methodological limitations inherent in quantifying the degree of treatment resistance. However, an alternative explanation is that there is no consistent association between brain structure and the number of trials of antidepressant medication without clinical response. The latter explanation is supported by the fact that so few brain regions correlated with MGH-S scores in the group-level regression analysis.

Hippocampal volume reductions have been reported in several TRD (and MDD) studies previously [9–12, 45]. Although no significant differences were identified in this region between TRD participants and healthy controls, hippocampal volume was found to negatively correlate with both the HDRS_17_ and MADRS scores (see S1 File). Nonetheless, given the chronicity, severity and degree of treatment-refractoriness of the participants’ illnesses, the absence of hippocampal volumetric differences between the groups was somewhat surprising. The extensive treatment history all TRD patients, or inclusion of TRD participants with mild illness severity (or remission) at the time of scanning in the present study may account for the failure to replicate these between-group hippocampal volume reductions as a number of studies suggest a correlation between hippocampal volume and antidepressant treatment/decreasing depressive symptoms [46–50].

Caudate volume reductions have been reported in previous studies of depression [45, 51–54] and negative correlations between caudate volume and HDRS_17_ scores have been reported previously [51, 55]. In addition, decreased connectivity between the precuneus/posterior cingulate cortex and the bilateral caudate has been reported in early depression [56]. Although some studies did not find significant differences in caudate volume between groups, there are various factors that could explain this discrepancy including, for example, younger study populations, lower illness severity and the exclusion of subjects with cerebrovascular risk factors [55, 57, 58]. It is particularly interesting that the dorsal striatum (including the caudate) and not the ventral striatum was found to have reduced volume in the TRD group since the dorsal striatum is generally implicated in learning stimulus-response associations whereas the ventral striatum is involved with reward and motivation, particularly the prediction of reward [59].

The decrease in periventricular grey matter is linked with ventricular enlargement. Visual inspection of the raw MR images showed that several participants had remarkably large ventricles, including a few controls. Ventricular enlargement is more commonly associated with aging but, as there were no significant differences in age between the groups, it is most likely due to MDD diagnosis. Elkis, Friedman [60] reported, through a meta-analysis, that ventricular volumes were larger in patients with mood disorders than healthy controls, but smaller than in patients with schizophrenia. However, a later review by Soares and Mann [61] could not find evidence for ventricular enlargement in mood disorders. In an early structural study by Andreasen, Olsen [62], patients with schizophrenia and ventricular enlargement typically had more “negative” symptoms, the symptoms more commonly associated with depression such as anhedonia, and, similarly, patients with schizophrenia and small ventricles had predominantly “positive” symptoms. It is, therefore, possible that ventricular enlargement may be more strongly associated with motor symptoms and loss of hedonic responsiveness. However this association requires further investigation as neither the “psychomotor retardation” question within the HDRS_17_ score nor the “loss of pleasure” question within the BDI score correlated with CSF volume in this study.

Reduced habenula volume has also been reported, albeit inconsistently, in previous studies of patients with mood disorders [63, 64]. This reduction may be specific to mood disorder as reductions in this area have not been identified in studies of schizophrenia or post-traumatic stress disorder [63, 65]. Identification of a reduced habenular volume is interesting as it contains strong connections with both the limbic forebrain, consistently implicated in TRD studies, and the midbrain/brainstem and it is functionally associated with depression-relevant emotions (anxiety) and physiological responses such as those to stress and reward processing [63]. In addition, deep brain stimulation has been attempted previously in the lateral habenula in patients with treatment resistant depression [66].

Predictions of symptom severity in individual subjects have been performed previously in participants with current symptoms of depression [21] and other disorders such as OCD, Alzheimer’s disease and mild cognitive impairment [67–69]. Mwangi *et al*. [21] reported the ability to predict scores on the BDI in MDD participants using whole brain grey matter images. Using a similar approach, in this study we were able to predict HDRS_17_ *and* MADRS scores. This may be because the participants in the present study were more treatment-resistant and had a wider range of symptom severity scores. In any event, both the HDRS_17_ and MADRS scores, which are based upon clinician ratings of descriptions of symptoms over the preceding week, combined with some quantification of mental state findings, could be significantly predicted based upon a single grey matter MR image. When predicting symptom severity, adding feature selection to the process conferred little additional benefit. As some participants, despite having an extensive treatment history, were in remission at the time of scanning, it is a remarkable and novel finding that it was possible to accurately predict state measures of depression in individual subjects using structural measures.

### Study Limitations

There are several study limitations to note. First, although the number of subjects in this study is comparable with similar studies, replication in a larger study population would be desirable. Second, the TRD participants were taking a range of antidepressant medications at the time of scanning and it remains unclear to what extent this may influence results. Sapolsky [70] has argued that it is unlikely that the often observed grey matter reductions in MDD are a consequence of antidepressant medication as there is evidence for the contrary, antidepressant-induced neurogenesis, with no clear rationale for reductions. We observed some small regions of increased grey matter in the posterior cerebellum and middle frontal gyrus and it is possible these represent medication effects that merit further investigation. Current medication status is also, theoretically, a potential confound when predicting symptom severity scores. However, Table 2 shows that there is no obvious link between current medication and symptom severity. Despite the TRD patients, by definition, having an extensive medication history, there were very few specific brain regions that correlated with the MGH-S scores. The insula was the only region of interest that was linked with medication exposure, implying that previous medication exposure is not strongly associated with widespread regional grey matter loss. This is despite the participants in this study having experienced far greater lifetime exposure to antidepressant medications (see MGH-S scores) than those in other studies. Finally, as the TRD group was recruited with a past or present diagnosis of chronic MDD, the differences between the two groups may not have been as distinct as other studies. However, this enhanced range of symptom severity may actually have been an advantage in the prediction of symptom severity.

**Table 2:** Treatment Resistance, State Illness Severity and Current Medication. No patients had psychotic symptoms and quetiapine was prescribed as an augmentation agent for antidepressants [71], similar to the long established use of lithium, L-tryptophan and tri-iodothyronine in treatment resistant depression. No obvious relationships between current medication and treatment resistance/state illness severity were present. ‘mg’ indicates total dose per day, ‘mcg’ total micrograms per day.

HDRS_17_	Primary Anti-depressant	Secondary Anti-depressant	Primary Augmentation	Secondary Augmentation	Anti-psychotic Medication
21	fluoxetine (60 mg)	mirtazapine (45 mg)	lithium (900 mg)		
4	venlafaxine (525 mg)	mirtazapine (45 mg)			
11	sertraline (100 mg)	trazodone (200 mg)			quetiapine (300 mg)
8	venlafaxine (300 mg)		lithium (200 mg)		
16	phenelzine (60 mg)		L-Tryptophan (3000 mg)	lithium (1000 mg)	quetiapine (75 mg)
29					chlorpromazine (150 mg)
19	venlafaxine (300 mg)		L-Tryptophan (6000 mg)		
21	fluoxetine (100 mg)	trazodone (150 mg)			
18	venlafaxine (525 mg)	trazodone (150 mg)			
18	isocarboxazid (70 mg)				
24	sertraline (300 mg)	trazodone (300 mg)	tri-iodothyronine (20 mcg)		quetiapine (800 mg)
12	venlafaxine (75 mg)				
14	tranylcypromine (70 mg)				
19	isocarboxazid (40 mg)				quetiapine (75 mg)
13	sertraline (100 mg)				quetiapine (100 mg)
16	sertraline (200 mg)				quetiapine (300 mg)
14	venlafaxine (300 mg)				quetiapine (200 mg)
18	venlafaxine (225 mg)				
14	citalopram (60 mg)				
13	citalopram (10 mg)				

## Conclusions

To summarise, it was possible to use grey matter MR images to predict diagnostic status when comparing TRD subjects and healthy controls. Although it was not possible to predict the level of treatment-resistance in individual TRD participants, the insula was identified as a region of interest. These results provide encouragement that machine learning methods can increase the understanding of the neurobiology of TRD.

## Supporting Information

S1 File Supporting Information and Severity Score Prediction Information. (DOC)

S1 Fig The best fit lines for whole brain severity score predictions (left: HDRS_17_ predictions, right: MADRS predictions). (TIF)

S2 Fig The brain regions which were identified as the most predictive during the whole brain severity score predictions (left: HDRS_17_ predictions, right: MADRS predictions). (TIF)

S3 Fig The best fit line for the prediction of the BDI score using thresholded multiple linear regression feature selection and grey matter images. (TIF)

S4 Fig Group-level positive correlations between grey matter and HAM_D (top) and MADRS (bottom). (TIF)

S5 Fig Group-level negative correlations between grey matter and HAM_D. (TIF)

S6 Fig Group-level negative correlations between grey matter and MADRS. (TIF)

S7 Fig Group-level grey matter decreases in patients with TRD with increased BDI scores. (TIF)

S8 Fig Group-level grey matter decreases in patients with TRD with increased MGH-S scores. (TIF)

S9 Fig The best fit line for the correlation between the MGH-S scores and the volume of grey matter contained within a 5mm sphere centred within the mid-insulae (left (top), right (bottom)). (TIF)

S1 Data Supplemental data. (ZIP)
