# Supplementary material for: Structural MRI-Based Predictions in Patients with Treatment-Refractory Depression (TRD)
Source: PLoS One. 2015 Jul 17;10(7):e0132958. doi: 10.1371/journal.pone.0132958 (PMC4506147; doi:10.1371/journal.pone.0132958)
Supplement: S1 File — (DOC) [file pone.0132958.s001.doc]

**Supporting Information**

**Supporting Introduction**

To date, the study by Mwangi and colleagues remains the only published study that has applied machine learning to predict individual severity scores in depression, but there are several studies that have calculated group-level correlations with severity scores. The majority of these studies have performed correlation analyses with HDRS_17_ scores. Vakili *et al.* [1] found that bilateral hippocampal volume was negatively correlated with HDRS_17_ scores in males, but not females. Studies have also identified the caudate, bilateral dorsal prefrontal, bilateral medial frontal, inferior and superior frontal, orbitofrontal and cingulate cortices, bilateral temporal fusiform gyrus, occipital lobe, inferior temporal gyrus, amygdala / parahippocampal gyrus and postcentral gyrus as brain regions that are negatively correlated with HDRS_17_ scores in MDD subjects [2, 3]. In addition, positive correlations between HDRS_17_ scores and the occipital cortex and cerebellum were identified [2]. The only study that reported significant correlations with BDI scores found that decreased grey matter volume in the right planum temporale correlated with increased BDI scores [4]. However, Kim *et al.* [5] could not find a significant correlation between BDI scores and volume estimates within a number of a priori regions of interest. To our knowledge, no study has reported correlations between structural MRI and the MADRS scores in MDD patients.

**Supporting Methods**

One feature selection method used for the RVR prediction involved multiple linear regressions as implemented in the SPM toolbox and the method for optimising the feature selection was analogous to the classification procedure. In addition, multivariate feature selection (Recursive Feature Elimination, RFE [6-9]) was also tested to see if it could improve the prediction.

Overfitting, the inability to generalise to novel data, is a major potential issue when using RFE. However, this was not found to be an issue in this study as the results were able to be replicated on novel data during cross-validation. This issue may have been mitigated by reducing the number of folds during the inner *N*-fold cross-validation process optimising the RVR and feature selection parameters. All RFE results used 3-fold cross-validation during optimisation of the training data and LOOCV on the outer cross-validation loop, as this does not affect overfitting and maximises the data available to the training set.

**Supporting Results**

**Individual Subject Severity Score Predictions using Feature Selection**

Using feature selection to attempt to improve symptom severity score prediction above that achieved with whole brain predictions produced mixed results. When using thresholded multiple linear regression for feature selection, only the predicted BDI score was significantly correlated with the corresponding true score when using grey matter and a linear kernel (RMSE = 9.9218, MAE = 7.6304, R = 0.47901, p = 0.03, shown in S3 Fig.).

Although this finding was significant when using thresholded multiple linear regression to perform feature selection, the linear trends identified were unimpressive. Furthermore, the number of voxels and the brain regions identified in the predictions were too sparse and inconsistent to achieve confidence in these results. Therefore, a multivariate feature selection approach, RFE, was investigated to see if it could provide more reliable findings. Using RFE on grey matter images provided the *opposite* result to those found using thresholded multiple linear regression and a linear kernel, namely, the BDI prediction was not significant, but the HDRS_17_ (RMSE = 4.5583, MAE = 3.6615, R = 0.55464, p = 0.01) and MADRS (RMSE = 6.6423, MAE = 5.9254, R = 0.54238, p = 0.01) predictions were significant.

**Group-level Correlations (Severity Scores)**

Multiple linear regressions were performed on TRD participants’ grey matter images to see which regions positively, or negatively, correlated with symptom severity scores. As higher scoring in each of these symptom severity ratings indicates more severe symptoms of depression, it would be expected that negative correlations (whereby more severely depressed patients have reduced grey matter volume) would be more likely than positive correlations given the majority of the between-group differences in this study (and within the wider MDD literature). All results shown are p<0.05, whole brain level significance.

The MADRS and HDRS_17_ regressions gave similar results. Increased grey matter with increasing severity scores were found in the posterior cingulate gyrus and thalamus (shown in S4 Fig. and Tables D and E in S1 File). Also the anterior cingulate gyri and basal ganglia were identified as having increased grey matter with increased MADRS score (S4 Fig. and Table E in S1 File). Decreases (negative correlations) were found with the hippocampus, medial orbitofrontal cortex and periventricular grey matter (shown in S5 Fig. and Table D in S1 File for HDRS_17_ and S6 Fig. and Table E in S1 File for MADRS).

The grey matter correlation with BDI scores identified *decreased* grey matter in the cingulate sulcus and the lateral orbitofrontal area. The significant regions from the negative correlations are shown in S7 Fig. and Table F in S1 File.

**Supplementary References**

Table A: MNI coordinates of each cluster of grey matter identified using thresholded t-test feature selection with a Gaussian SVM. The number of resampled voxels contained in each cluster was calculated through a custom cluster-identification code.

| Region | MNI coordinates | | | Resampled voxels per cluster |
| --- | --- | --- | --- | --- |
|  | *x* | *y* | *z* |  |
| Insula | -46 | 16 | -14 | 346 |
|  | 46 | 9 | -1 | 122 |
|  | 41 | -1 | -11 | 36 |
| Caudate/periventricular grey matter | -13 | 14 | 15 | 271 |
|  | 19 | -15 | 27 | 41 |
|  | 14 | 21 | 1 | 37 |
| Posterior cingulate | -11 | -42 | 40 | 36 |
|  | 17 | -36 | 42 | 30 |
| Superior temporal gyrus | 26 | 13 | -25 | 170 |
|  | -30 | 14 | -28 | 45 |
| Inferior frontal gyrus | -45 | 10 | 20 | 156 |
|  | -49 | 29 | 17 | 33 |
|  | 42 | 25 | 5 | 33 |
|  | 36 | 26 | -7 | 32 |
|  | -31 | 42 | 14 | 32 |
| Middle temporal gyrus | -48 | -16 | -12 | 120 |
|  | -57 | -65 | 18 | 90 |
|  | -68 | -34 | 0 | 67 |
|  | -41 | -72 | 14 | 60 |
|  | 57 | -23 | -3 | 48 |
|  | 69 | -42 | -10 | 32 |
| Medial orbital gyrus | -6 | 29 | -29 | 101 |
| Occipital lobe | -15 | -101 | -14 | 94 |
| Superior frontal gyrus | -8 | -14 | 49 | 93 |
|  | -18 | -12 | 61 | 45 |
| Medial frontal gyrus | -23 | 10 | 46 | 89 |
|  | -33 | -5 | 48 | 74 |
|  | 36 | 6 | 33 | 39 |
|  | 30 | 13 | 47 | 38 |
| Cerebellum | 7 | -81 | -42 | 51 |
|  | -6 | -83 | -38 | 35 |
| Inferior parietal lobe | -50 | -37 | 54 | 50 |
|  | -50 | -33 | 29 | 39 |
| Thalamus | 10 | -10 | 16 | 48 |
|  | -9 | -10 | 16 | 35 |
| Superior parietal lobe | 27 | -49 | 46 | 48 |
|  | -41 | -68 | 34 | 36 |

Table B: MNI coordinates of each cluster of grey matter identified in the VBM analysis (significance defined as p<0.05 at a whole brain, family-wise error corrected level, with the simultaneous requirements for voxel threshold and minimum cluster extent).

Decreases (patient < control):

| Region | MNI coordinates | | | Resampled voxels per cluster | T-score |
| --- | --- | --- | --- | --- | --- |
|  | *x* | *y* | *z* |  |  |
| Caudate/periventricular grey matter | -12 | 22 | -2 | 1135 | 3.10 |
|  | 16 | 22 | 2 |  | 2.39 |
| Insula | -46 | 18 | -10 | 2330 | 2.77 |
|  | -44 | 8 | -14 |  | 2.57 |
|  | 46 | 8 | 0 | 1349 | 2.54 |
|  | 50 | 12 | 10 |  | 2.97 |
| Habenula region | -2 | -26 | 2 | 84 | 2.08 |
|  | 6 | -30 | 4 |  | 2.03 |
| Amygdala | 14 | -2 | -20 | 76 | 2.23 |
| Anterior cingulate | 2 | 32 | 2 | 85 | 1.92 |
| Posterior cingulate | -16 | -22 | 42 | 715 | 2.10 |
|  | 16 | -36 | 42 | 70 | 2.80 |
| Midbrain | -14 | -16 | -18 | 103 | 2.21 |
| Inferior parietal lobe | -56 | -32 | 30 | 203 | 2.27 |
| Angular gyrus | -58 | -66 | 22 | 181 | 2.90 |
| Occipital lobe | -18 | -102 | -16 | 174 | 2.84 |
| Medial orbital gyrus | -6 | 32 | -32 | 264 | 2.83 |
| Inferior parietal lobule | -52 | -36 | 54 | 339 | 2.73 |
|  | 70 | -24 | 26 | 108 | 2.54 |
|  | -42 | -60 | 42 | 93 | 1.93 |
| Middle temporal gyrus | -40 | -62 | 4 | 177 | 2.07 |
|  | 68 | -48 | -8 | 75 | 2.41 |
|  | 58 | -24 | -2 | 134 | 2.40 |
|  | -46 | -46 | 4 | 76 | 2.33 |
| Inferior frontal gyrus | -50 | 30 | 14 | 77 | 2.35 |
| Middle frontal gyrus | 42 | 6 | 32 | 77 | 2.21 |
| Superior parietal lobule | 8 | -76 | 56 | 100 | 2.07 |

Increases (patient > control):

| Region | MNI coordinates | | | Resampled voxels per cluster | T-score |
| --- | --- | --- | --- | --- | --- |
|  | *x* | *y* | *z* |  |  |
| Superior parietal lobe | 24 | -48 | 44 | 85 | 2.97 |
| Superior frontal gyrus | -22 | 12 | 46 | 196 | 2.90 |
|  | -20 | -14 | 58 | 74 | 2.77 |
| Middle frontal gyrus | -36 | -4 | 50 | 100 | 2.73 |
|  | 28 | 12 | 44 | 107 | 2.55 |
| Posterior cerebellum | 6 | -80 | -40 | 461 | 2.49 |

Table C: MNI coordinates of each cluster of grey matter negative correlations with MGH-S scores (significance defined as p<0.05 at a whole brain, family-wise error corrected level, with the simultaneous requirements for voxel threshold and minimum cluster extent).

| Region | MNI coordinates | | | Resampled voxels per cluster | T-score |
| --- | --- | --- | --- | --- | --- |
|  | *x* | *y* | *z* |  |  |
| Insula | -58 | 16 | -2 | 139 | 2.91 |
|  | 46 | 18 | -4 | 154 | 2.11 |
| Superior parietal lobe | -32 | -60 | 64 | 1099 | 4.37 |
|  | 34 | -74 | 50 | 242 | 3.33 |
| Precentral gyrus | 14 | -22 | 76 | 1052 | 3.99 |
| Middle frontal gyrus | -38 | 22 | 54 | 878 | 3.58 |
|  | 20 | 18 | 66 | 101 | 3.36 |
|  | -24 | 34 | 54 | 329 | 3.32 |
| Occipital lobe | 20 | -64 | 26 | 2700 | 3.49 |
|  | -38 | -74 | -6 | 191 | 3.42 |
|  | 32 | -78 | 16 | 117 | 3.38 |
| Medial temporal gyrus | -50 | -66 | 4 | 109 | 3.38 |
| Cerebellum | -30 | -36 | -44 | 121 | 3.27 |
| Inferior frontal gyrus | 62 | 12 | 30 | 337 | 3.25 |

Table D: MNI coordinates of each cluster of grey matter correlations with HDRS_17_ scores (significance defined as p<0.05 at a whole brain, family-wise error corrected level, with the simultaneous requirements for voxel threshold and minimum cluster extent).

Positive correlations:

| Region | MNI coordinates | | | Resampled voxels per cluster | T-score |
| --- | --- | --- | --- | --- | --- |
|  | *x* | *y* | *z* |  |  |
| Posterior cingulate | -6 | -26 | 36 | 1074 | 3.81 |
| Thalamus | 6 | -18 | 8 | 755 | 3.22 |
|  | -4 | -18 | 8 | 76 | 2.56 |
| Parahippocampal gyrus | 10 | -38 | 0 | 755 | 3.70 |
| Inferior parietal lobe | -36 | -56 | 24 | 224 | 5.91 |
|  | -52 | -44 | 36 | 160 | 4.96 |
| Superior parietal lobe | 44 | -46 | 36 | 869 | 4.82 |
|  | -22 | -54 | 42 | 117 | 3.96 |
| Postcentral gyrus | 34 | -24 | 48 | 454 | 3.38 |
|  | -42 | -24 | 54 | 233 | 2.89 |
| Superior frontal gyrus | 14 | 8 | 56 | 105 | 2.79 |
| Fusiform gyrus | -48 | -20 | -16 | 102 | 3.83 |
|  | -32 | -48 | -16 | 669 | 1.92 |
| Superior temporal gyrus | -44 | -38 | 10 | 83 | 3.49 |

Negative correlations:

| Region | MNI coordinates | | | Resampled voxels per cluster | T-score |
| --- | --- | --- | --- | --- | --- |
|  | *x* | *y* | *z* |  |  |
| Hippocampus/  periventricular grey matter | 22 | -38 | 8 | 806 | 3.40 |
|  | -26 | -44 | 2 |  | 2.36 |
|  | 0 | -10 | 18 |  | 2.98 |
| Medial orbitofrontal cortex | 4 | 46 | -4 | 1387 | 2.81 |
| Superior parietal lobe | -28 | -46 | 72 | 104 | 4.54 |
| Postcentral gyrus | -62 | -26 | 46 | 77 | 3.43 |
| Superior frontal gyrus | 6 | 0 | 72 | 1438 | 4.46 |
|  | 20 | 44 | 50 | 240 | 3.84 |
| Occipital lobe | -16 | -68 | 14 | 1991 | 3.82 |
| Angular gyrus | 56 | -66 | 34 | 171 | 2.63 |
| Middle frontal gyrus | 40 | 28 | 50 | 266 | 3.37 |
| Midbrain | -10 | -14 | -18 | 676 | 3.29 |
|  | 8 | -14 | -20 | 142 | 3.13 |

Table E: MNI coordinates of each cluster of grey matter correlations with MADRS scores (significance defined as p<0.05 at a whole brain, family-wise error corrected level, with the simultaneous requirements for voxel threshold and minimum cluster extent).

Positive correlations:

| Region | MNI coordinates | | | Resampled voxels per cluster | T-score |
| --- | --- | --- | --- | --- | --- |
|  | *x* | *y* | *z* |  |  |
| Posterior cingulate | -6 | -26 | 36 | 1298 | 4.16 |
| Thalamus/basal ganglia | 6 | -16 | 8 | 740 | 3.65 |
|  | -6 | -18 | 6 | 580 | 3.51 |
|  | -18 | 8 | 4 |  | 2.62 |
|  | 20 | 8 | 6 | 119 | 2.46 |
| Anterior cingulate | -6 | 30 | 10 | 110 | 3.13 |
| Superior parietal lobe | 44 | -44 | 34 | 848 | 5.17 |
| Inferior parietal lobe | -38 | -54 | 26 | 229 | 4.80 |
|  | -42 | -32 | 34 | 159 | 2.48 |
| Postcentral gyrus | -36 | -26 | 48 | 344 | 4.34 |
| Precentral gyrus | -50 | -4 | 22 | 243 | 2.97 |
|  | 50 | -4 | 22 | 297 | 3.47 |
| Inferior temporal gyrus | -46 | -14 | -18 | 115 | 4.19 |
| Middle frontal gyrus | 32 | 36 | 14 | 84 | 2.50 |

Negative correlations:

| Region | MNI coordinates | | | Resampled voxels per cluster | T-score |
| --- | --- | --- | --- | --- | --- |
|  | *x* | *y* | *z* |  |  |
| Hippocampus/  periventricular grey matter | 22 | -38 | 8 | 12541 | 3.86 |
|  | -26 | -44 | 2 |  | 2.43 |
|  | 0 | -6 | 18 |  | 3.70 |
| Medial orbitofrontal cortex | 10 | 54 | -2 | 12188 | 3.78 |
| Posterior cingulate | -16 | -28 | 44 | 97 | 2.06 |
| Precentral gyrus | -18 | -26 | 68 | 354 | 2.76 |
|  | 4 | -30 | 72 | 738 | 3.66 |
| Middle temporal gyrus | -60 | 6 | -18 | 293 | 3.79 |
|  | -48 | 6 | -44 | 113 | 2.27 |
| Inferior temporal gyrus | -56 | -30 | -30 | 120 | 2.28 |
| Superior temporal gyrus | 52 | -28 | 14 | 191 | 2.22 |

Table F: MNI coordinates of each cluster of grey matter negative correlations with BDI scores (significance defined as p<0.05 at a whole brain, family-wise error corrected level, with the simultaneous requirements for voxel threshold and minimum cluster extent).

| Region | MNI coordinates | | | Resampled voxels per cluster | T-score |
| --- | --- | --- | --- | --- | --- |
|  | *x* | *y* | *z* |  |  |
| Subgenual cingulate/ lateral orbitofrontal area | 44 | 40 | -6 | 11276 | 5.44 |
|  | -40 | 44 | 0 |  | 4.61 |
|  | 0 | 30 | -6 |  | 3.12 |
| Superior frontal gyrus | 6 | 54 | 18 | 326 | 2.60 |
| Inferior temporal gyrus | -56 | -52 | 2 | 196 | 3.54 |
|  | 50 | -50 | -12 | 105 | 3.21 |
| Middle temporal gyrus | -32 | 6 | -40 | 131 | 3.39 |
| Cerebellum | 28 | -40 | -38 | 310 | 2.84 |
|  | -32 | -38 | -36 | 77 | 2.47 |
| Fusiform gyrus | 26 | -50 | -6 | 115 | 3.07 |
|  | 26 | -14 | -28 | 354 | 2.68 |
| Superior temporal gyrus | -36 | -36 | 14 | 922 | 3.01 |
|  | 52 | 12 | -20 | 264 | 2.08 |
|  | -42 | -32 | 6 | 145 | 1.84 |
