# Supplementary figures and images for: Structural MRI-Based Predictions in Patients with Treatment-Refractory Depression (TRD)
Source: PLoS One. 2015 Jul 17;10(7):e0132958. doi: 10.1371/journal.pone.0132958 (PMC4506147; doi:10.1371/journal.pone.0132958)

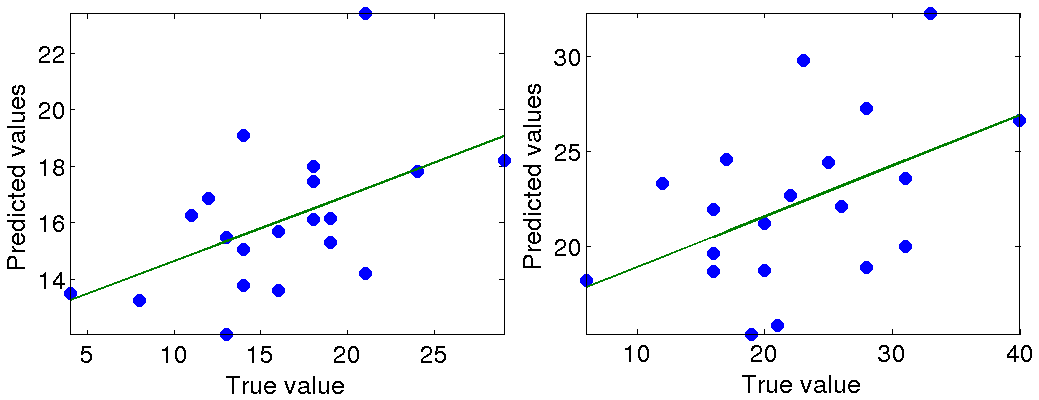

Supplement: S1 Fig — (TIF) [file pone.0132958.s002.tif]

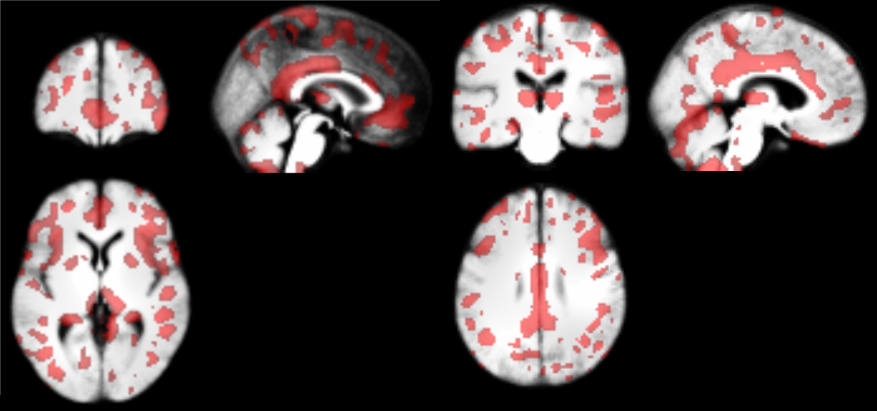

Supplement: S2 Fig — (TIF) [file pone.0132958.s003.tif]

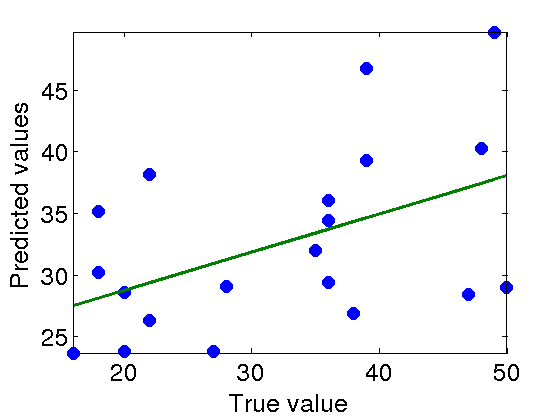

Supplement: S3 Fig — (TIF) [file pone.0132958.s004.tif]

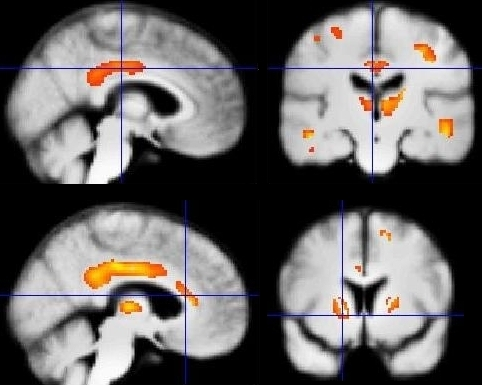

Supplement: S4 Fig — (TIF) [file pone.0132958.s005.tif]

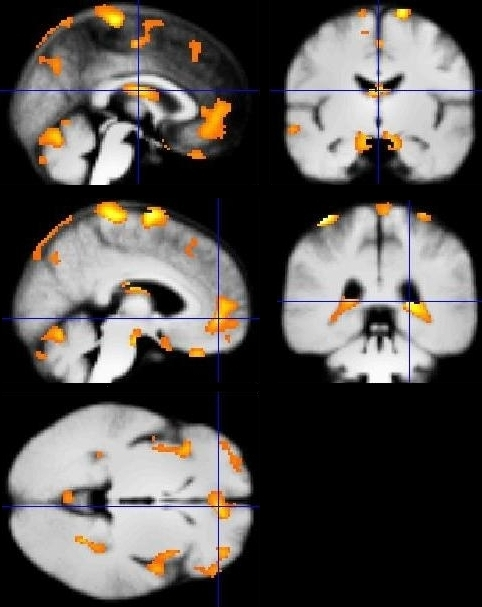

Supplement: S5 Fig — (TIF) [file pone.0132958.s006.tif]

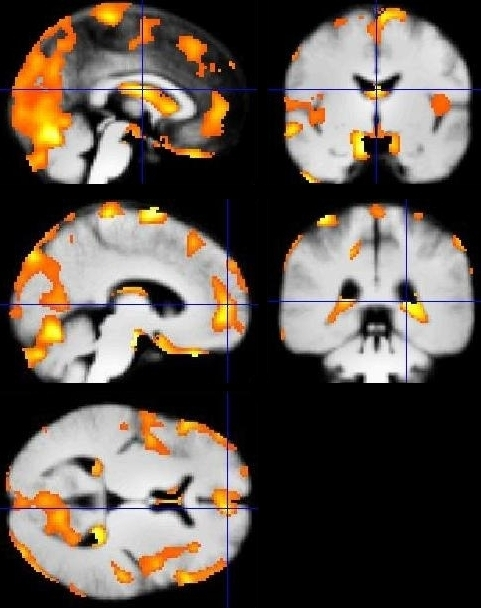

Supplement: S6 Fig — (TIF) [file pone.0132958.s007.tif]

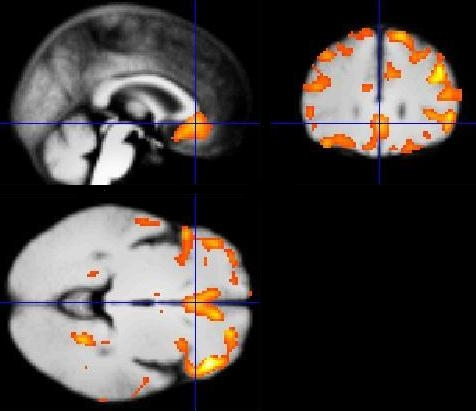

Supplement: S7 Fig — (TIF) [file pone.0132958.s008.tif]

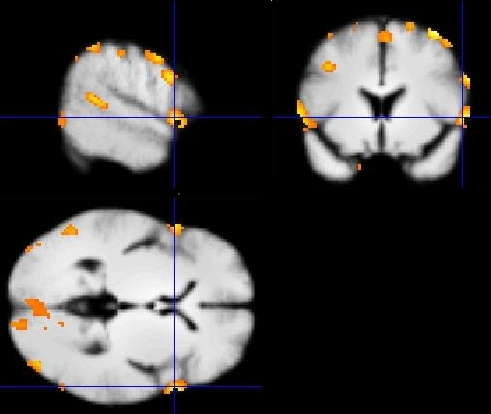

Supplement: S8 Fig — (TIF) [file pone.0132958.s009.tif]

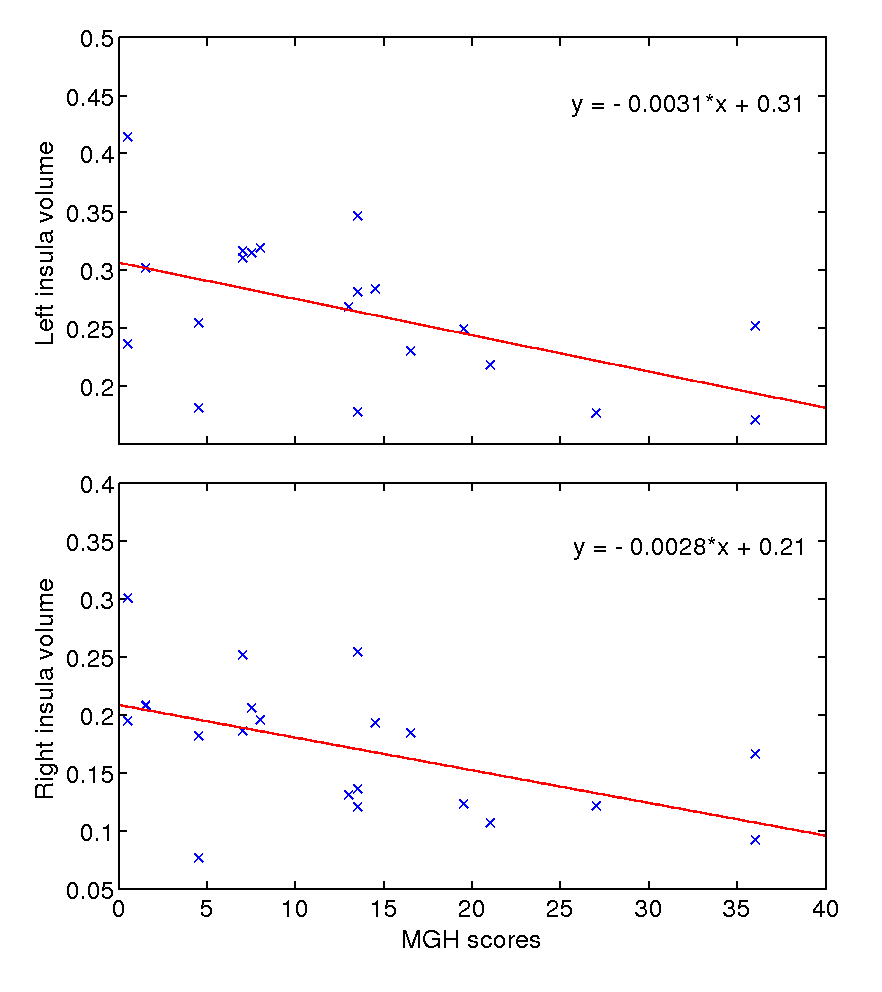

Supplement: S9 Fig — (TIF) [file pone.0132958.s010.tif]
